# Overview of current and future biologically based targeted therapies in head and neck squamous cell carcinoma

**DOI:** 10.1186/1758-3284-1-6

**Published:** 2009-03-02

**Authors:** Ajay Matta, Ranju Ralhan

**Affiliations:** 1 Department of Chemistry and Centre for Research in Mass Spectrometry, York University, 4700 Keele Street, Toronto, Ontario, M3J 1P3, Canada; 2 Joseph and Mildred Sonshine Family Centre for Head & Neck Disease, Mount Sinai Hospital, 600 University Avenue, Toronto, Ontario, M5G 1X5, Canada; 3 Department of Otolaryngology – Head and Neck Surgery, University of Toronto, 190 Elizabeth Street, Toronto, M5G 2N2, Ontario, Canada

## Abstract

Recent advances in genomics, proteomics, bioinformatics and systems biology have unraveled the complex aberrant signaling networks in cancer. The knowledge accrued has dramatically increased the opportunities for discovery of novel molecular targets for drug development. Major emphasis is being laid on designing new therapeutic strategies targeting multiple signaling pathways for more effective disease management. However, the translation of in vitro findings to patient management often poses major challenges that limit their clinical efficacy. Here we will discuss how understanding the dysregulated signaling networks can explain the pitfalls in translating the laboratory findings from the bench-to-bedside and suggest novel approaches to overcome these problems using head and neck cancer as a prototype. The five year survival rates of HNSCC patients (about 50% at 5 years) have not improved significantly despite advancements in multimodality therapy including surgery, radiation and chemotherapy. Molecular targeted therapies with inhibitors of EGFR and VEGF either alone, or in combination with conventional treatments have shown limited improved efficacy. The key deregulated signaling pathways in head and neck squamous cell carcinoma (HNSCC) include EGFR, Ras, TGFβ, NFκB, Stat, Wnt/β-catenin and PI3-K/AKT/mTOR. The aberrant activities of these interrelated signaling pathways contribute to HNSCC development. In depth understanding of the cross-talks between these pathways and networks will form the basis of developing novel strategies for targeting multiple molecular components for more effective prevention and treatment of HNSCC.

## Introduction

Head and neck squamous cell carcinoma (HNSCC) is the sixth most common cancer accounting for over 500,000 new cases annually worldwide [1]. Despite improvement in treatment strategies involving surgery, radiotherapy (RT) and/or chemotherapy (CT), the prognosis of HNSCC patients in advanced stages (III/IV) remains largely unsatisfactory owing to loco-regional recurrence [2,3]. Randomized trials using CT (cisplatin/carboplatin alone, or in combination with 5-Fluorouracil (5-FU), methotrexate or paclitaxel and/or RT show increased loco-regional control or survival and prevent subsequent metastasis by eradicating occult metastasis, though the dose limiting toxicities or increased risk of cardiac failure in cancer patients limits their clinical utility [4-6]. Hence major thrust is being laid on development of molecular targeted therapies for HNSCCs.

Multiple epigenetic and genetic events, including the aberrant expression and/or function of regulators of cell cycle, growth and signaling, motility, apoptosis, angiogenesis and microRNAs are implicated in pathogenesis of HNSCCs and constitute plausible targets for therapy. Advances in epigenomics, genomics, proteomics, bioinformatics and integration of this knowledge have provided holistic understanding of signaling pathways and networks that regulate cellular functions, intra- and inter-cellular communication, and tumor-host interactions. The deregulation of signaling cascades including the EGFR, Ras, NFκB, Stat, Wnt/β-catenin, TGF-β, and PI3-K/AKT/mTOR pathways contributes to development of HNSCC [7]. Here, we will discuss how this emerging information on cross-talks between the different signaling pathways and networks can help to understand the limited efficacy of mono-targeted therapies for HNSCC. In turn, this knowledge can be harnessed for developing novel multiple molecular-targeted strategies for HNSCC treatment.

### Molecular Targeted Therapies for HNSCC

**Figure 1:**
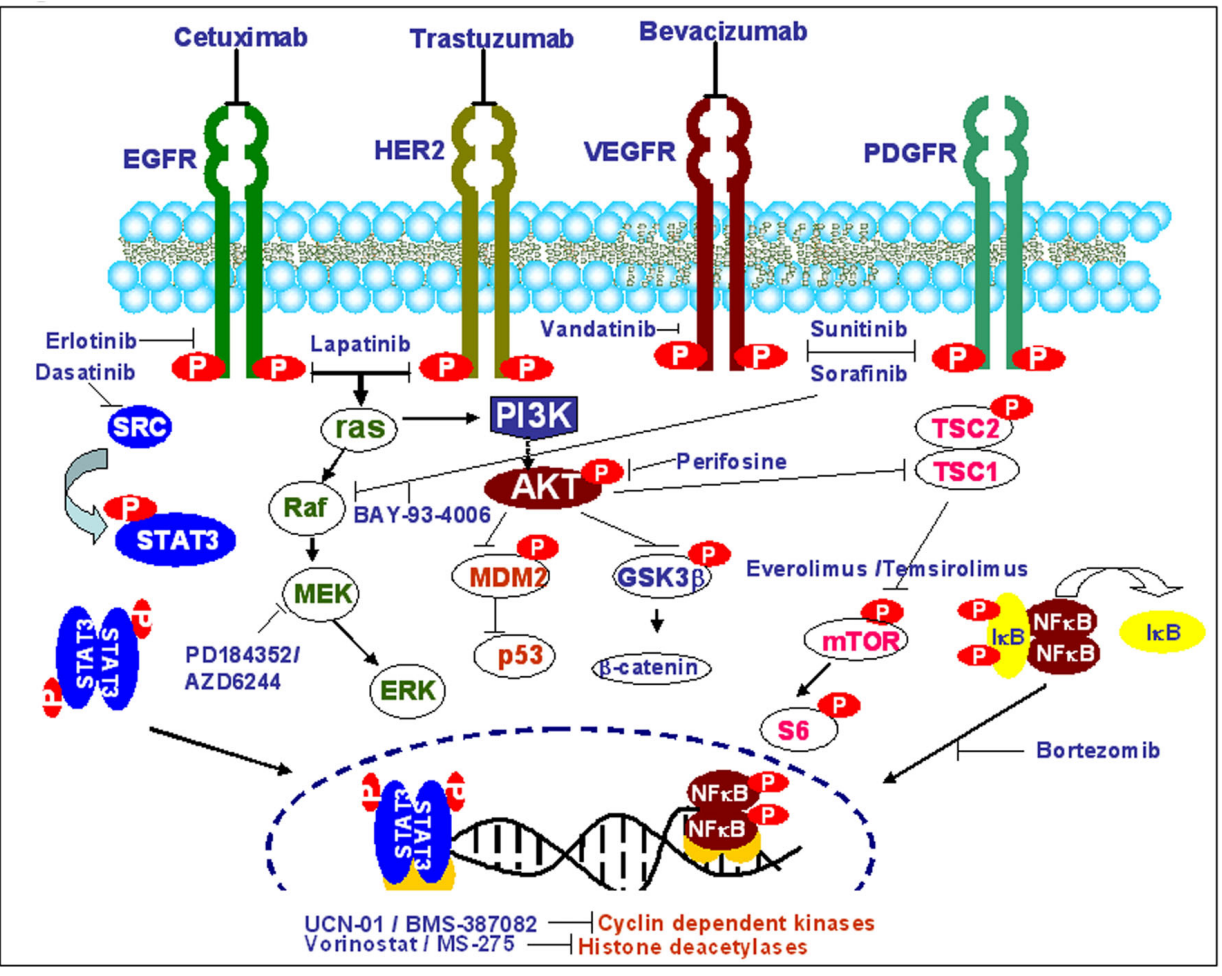
**Signaling pathways frequently deregulated in HNSCC, the molecular targets involved and their corresponding inhibitors as potential anticancer agents**.

Several molecular targeted therapies are currently being developed for HNSCC. The signaling pathways deregulated in HNSCC and the agents targeting key components are schematically represented in Figure 1. The clinical efficacies of these inhibitors targeting important pathways regulated by epidermal growth factor receptor (EGFR), vascular endothelial growth factor (VEGF) and AKT have been reviewed [8-14]. Large amount of preclinical in vitro and in vivo data have been obtained on the anti-proliferative properties of these inhibitors, both as single agents and combined with CT/RT. The inclusion of these agents in combined modality treatment regimes for early and/or advanced stage HNSCC is likely to increase therapeutic efficacy. Consequently, several targeted agents are under clinical trials in HNSCC, with many phase I/II studies already completed and some phase III studies in progress. The limited efficacies of these trials and unexpected toxicities in HNSCC patients have emphasized the difficulties of translating in-vitro findings to clinics for disease management.

### EGFR Inhibitors: Clinical Challenges

Activation of EGFR signaling is one of the mechanisms for resistance to RT and/or CT in HNSCC, making it the most plausible therapeutic target [15-17]. Upon ligand binding (EGF or TGF α), EGFR forms a homodimer or heterodimer with other members of the Erb family (Her2/neu, Erb3, Erb4) and activates downstream signaling cascades-Ras/Raf/MAPK and the PI3K/Akt/mTOR pathways (Figure 1). The activation of these signaling events is responsible for regulating key tumorigenic processes such as proliferation, inhibition of apoptosis, cell adhesion/motility, growth and survival. Monoclonal antibodies against the extra-cellular domain of EGFR, cetuximab, pertuzumab, panitumumab and trastuzumab, used as inhibitors in monotherapy have shown limited efficacy. In a phase I/II trial, combination of cetuximab with 5-FU and carboplatin/cisplatin showed increased survival with no cumulative toxicity in recurrent HNSCC [18]. Cetuximab acts as a tumor specific radiosensitizer [19,20]. EGFR inhibition by cetuximab significantly reduced tumor repopulation during fractionated RT in a xenografted human model of SCC [21]. In contrast, trials with chemoradiation (CRT) and cetuximab in HNSCC have shown adverse events and withdrawal of the trial [22]. Humanized antibodies- panitumumab and trastuzumab overcome the dose dependent toxicity of cetuximab but are less immunogenic and effective. Moreover, clinical trials of trastuzumab for HNSCC have reported cardiomyopathy in patients undergoing treatment. ErbB2 inhibition has been shown to activate mitochondrial apoptosis by modulating the ratio of BCL-xL and -xS, resulting in cardiomyopathy [23], but the exact mechanism of trastuzumab induced cardiomyopathy is still unclear. Unlike monoclonal antibodies, tyrosine kinase inhibitors (TKIs) such as gefitinib, erlotinib and lapatinib block the ATP pocket of EGFR, thereby inhibiting phosphorylation and down-stream signal transduction. A multicentric study showed that erlotinib was well tolerated in pretreated HNSCCs and prolonged disease free survival [24].

Although, EGFR overexpression is observed in more than 90% of HNSCCs, yet, only a subset of these tumors show a clinically meaningful response to EGFR inhibition [25,26]. Potential reasons for failure of response to EGFR inhibitors include: constitutive activation of the Ras/Raf/MAPK, STAT3 and PI3-K/AKT/mTOR signaling pathways independent of EGFR by other stimuli such as hypoxia, *Ras *activation or *PTEN *mutation and inhibition. The presence of EGFR variant III (EGFRvIII) in HNSCCs, is also responsible for constitutive activation of downstream signaling and resistance to EGFR inhibition by monoclonal antibodies [27]. Acquired resistance to cetuximab is accompanied by deregulation of EGFR internalization/degradation and subsequent EGFR dependent activation of HER3 [28]. EGFR inhibition by erlotinib/gefitinib is overcome by epithelial-mesenchymal transition [29]. Recently, Hadad et al., [30] proposed a novel mechanism for regulation of mesenchymal phenotype and resistance to erlotinib in HNSCC cells by Delta-crystallin enhancer binding factor 1. In addition, cross-talks between EGFR and cell adhesion molecules, cytokine receptors, ion channels and G protein coupled receptor (GPCR) lead to EGFR activation [31]. GPCR-EGFR cross-talk may play a role in development of HNSCC and account for limited efficacy of EGFR inhibitors in HNSCC. The aforementioned mechanisms might also explain why most clinical trials suggest no correlation between EGFR protein expression and response to EGFR inhibitors. Notably, favorable outcome has been associated with skin toxicity or presence of shorter EGFR intron 1 cytosine-adenine repeats [32]. As yet, there are no proven molecular predictors of response to EGFR targeted antibodies [33]; search for biomarkers should be extended to EGFR-activation status and key components of downstream pathways. Tumor signaling pathway components that work synergistically with EGFR or compensate for the loss of EGFR-initiated signaling are likely to be ideal targets for multi-targeted therapy. Erb family-targeted and Src family-targeted agents are in clinical development [34].

### VEGF Inhibitors

Increased expression of VEGF and its receptors in HNSCCs, underscores the importance of VEGF pathway in angiogenesis and survival of tumor cells under hypoxic conditions [35,36]. VEGF expression is regulated by hypoxia-inducible factor -1α (HIF-1α) -dependent and independent processes, both of which involve PI3-K and AKT. Bevacizumab, a humanized VEGF monoclonal antibody, not only inhibits angiogenesis, but also facilitates the increased delivery of chemotherapeutic agents by decreasing microvascular permeability and decreasing intratumor pressure. But, single-agent anti-angiogenic drugs have not shown activity in unselected HNSCC patients, with a response rate of less than 4%. On the other hand, combinations of bevacizumab with erlotinib showed a response rate of 14.6%. Studies of bevacizumab with CT (phase III Eastern Cooperative Oncology Group [ECOG] trial) and in combination with CRT are currently in progress [37]. However, HNSCCs show inter-tumoral angiogenic heterogeneity; in-depth understanding of the variability of angiogenic phenotype within a given HNSCC is important for designing cytokine targeted anti-angiogenic therapies.

### Multikinase Inhibitors

Sorafenib, an oral multikinase inhibitor, targets serine/threonine Raf-1 kinase and receptor tyrosine kinases (RTKs)- VEGFR, PDGFR, KIT, and Flt3. Phase II trials in recurrent or metastatic HNSCC patients showed that sorafenib was well tolerated with modest anticancer activity comparable to monotherapy [38]. BIBF 1120, targets VEGF, PDGF, FGF receptor and src family of tyrosine kinases (Src, LcK, Lyn). Vandetanib (ZD6474), an inhibitor of VEGFR, EGFR, and rearranged during transfection (RET) tyrosine kinases is being tested in HNSCC as monotherapy and also in combination with CT [39,40]. Dasatinib (BMS-354825) is a synthetic, small molecule inhibitor of Src family kinases, also inhibits protein tyrosine kinases: bcr-abl, EphA2, PDGF-β [41]. But, a major challenge in development of multikinase inhibitors is the rapid evolution of mutant inhibitor resistant kinases, therefore, appropriate multi-targeted inhibitors or combinations need to be planned in advance of clinical application.

### PI3-K/AKT/mTOR pathway Inhibitors

Uncontrolled activation of the PI3-K/Akt/mTOR pathway contributes to the development and progression of HNSCC and is an important target to counteract resistance to RT and/or CT [42]. *PTEN *deletions and 'hot-spot' mutations of the *PI3K *gene have been shown to possess transforming capacity in vitro and in vivo, hence restoration of mutated or absent PTEN activity might be a target for AKT inhibition. Protease inhibitors downregulate the phosphorylation and expression of active PI3-K, that is responsible for radioresistance in HNSCC. Akt activation is a possible mechanism of resistance to EGFR inhibitors, therefore, the combination of AKT inhibitors and anti-EGFR agents may be useful in effective management of HNSCC. The mammalian target of rapamycin, commonly known as mTOR regulates cell growth, proliferation, motility, survival, protein synthesis, and transcription. Rapamycin derivatives such as everolimus, temserolimus and deforolimus are potent inhibitors of mTOR and do not share the problems of poor solubility and chemical stability of rapamycin. A clinical trial using cisplatin and everolimus (RAD-001) in HNSCC is in progress [43]. However, not all HNSCCs have activated PI3-K/Akt/mTOR pathway, hence molecular signatures need to be developed to define patients that may benefit from inhibitors of this pathway. Moreover, mTOR inhibition blocks the natural negative feedback on insulin-like growth factor-1 receptor (IGF-1R) signaling impinging on PI3-K. This results in increased PI3-K and Akt activation which could potentially counteract the inhibition of mTOR. Dual inhibition of both IGF-1R signaling or TKIs, and mTOR may result in a superior anti-proliferative effect over each single strategy in HNSCCs [44,45]. The major limitation of mTOR inhibitors in clinical trials is their dose limiting toxicity. Hence, natural products, such as curcumin, which hit multiple cellular targets including mTOR, in combination with mTOR inhibitors may reduce the toxic side effects and augment the clinical efficacy.

### Novel Biological Targeted Agents for HNSCC

Biological agents, pemetrexed and enzastaurin, an oral protein kinase C beta (PKCbeta) inhibitor, are showing considerable promise and no unexpected toxicities in phase I trials in combination with cisplatin [46,47]. Aurora kinase A (AURKA) inhibitor and paclitaxel in combination are also under investigation for HNSCC management [48]. Among the new COX-2 inhibitors, Salvianolic acid B (Sal-B) has shown promise for HNSCC prevention and treatment [49]. Geldanamycin analogues have demonstrated potent inhibition of Hsp90 demonstrating significant anti-tumor activity in both cell culture and animal studies. Bortezomib inhibits activation of NFκB and sensitizes these cells to chemotherapy, radiation, or immunotherapy without added toxicities [50-53]. Histone deacetylases (HDACs) are enzymes that regulate the acetylation of histone proteins and non-histone proteins including p53, p21, NFκB. Altered expressions of HDACs have been reported in several human malignancies including head and neck cancer. Inhibitors of HDAC such as suberoylanilide hydroxamic acid have been shown to induce growth arrest, differentiation and promote apoptosis of HNSCC cell lines [54]. HDAC inhibitors target NFκB and also increase radiosensitivity and thus may be tried in future trials.

### Challenges in Molecular Targeted Therapies for HNSCC

**Figure 2:**
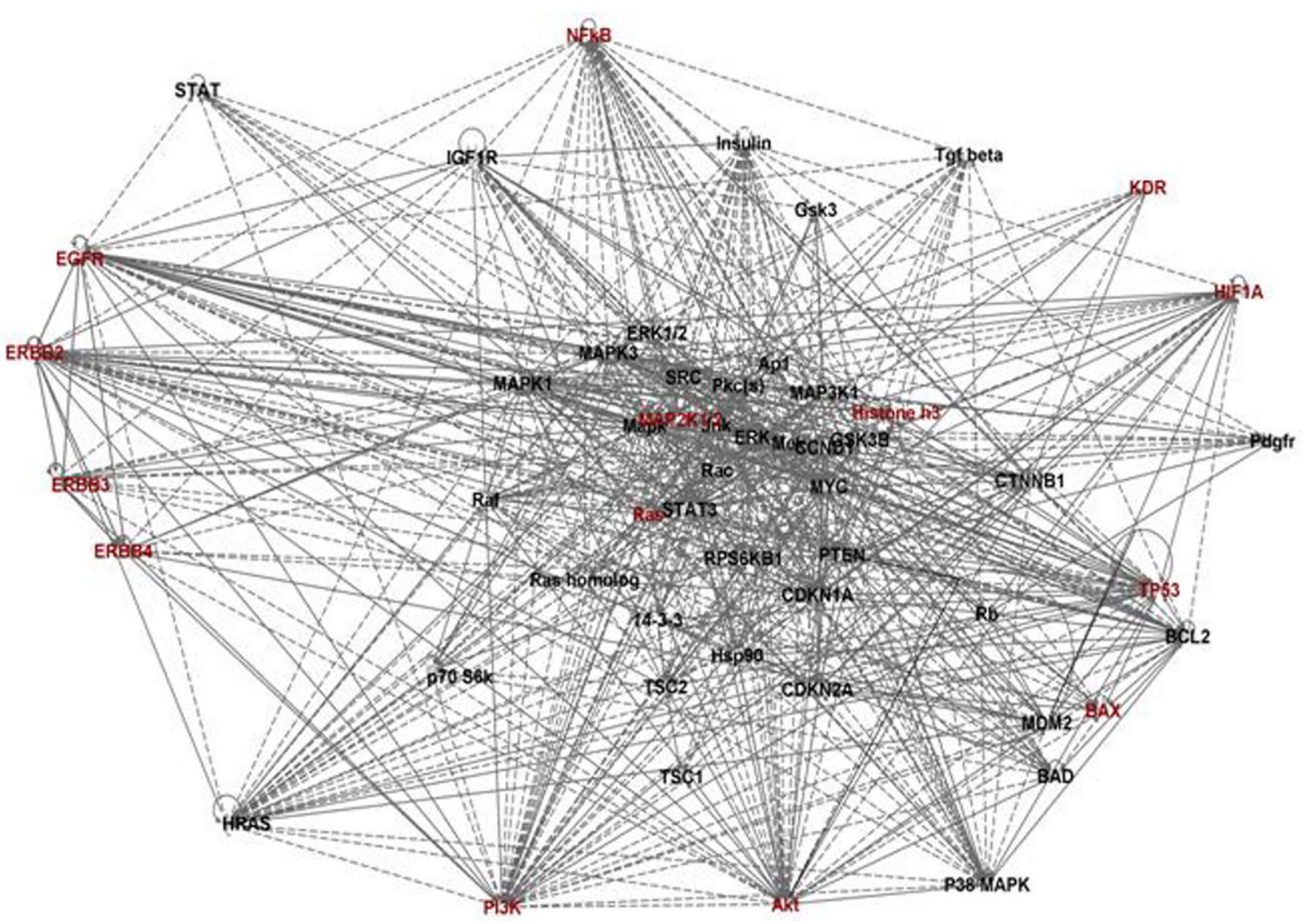
**Network analysis using Ingenuity pathway analysis (IPA) software**. This figure depicts the merged networks of proteins/molecules targeted for novel therapies in HNSCCs. These proteins/molecules form a complex network responsible for survival and proliferation of cancer cells. Therefore, targeting this complex network with single agents or monotherapy is often a failure as blocking a single pathway triggers alternate signaling cascades to activate downstream targets for survival. The bold lines show a direct association among different proteins/molecules while the dashed lines represent indirect regulation or association among these proteins. As shown, all members of Erb family are directly associated; hence dowregulating/blocking one of the members does not block downstream signaling via Ras/Raf/Mapk and PI3K/Akt/mTOR pathway completely. Also, as shown in figure, these pathways are also being regulated by Insulin, TGF-β and KDR (VEGFR), PDGFR and HIF1α.

The complexity of the aberrant signaling in HNSCC (Figure 1 &2) explains why interfering with only single steps in these pathways have not shown marked clinical response in HNSCC patients. HNSCC cells have the ability to exploit diverse signaling pathways for growth advantage, cell survival and evasion of apoptosis. In fact, some of these processes may even be facilitated by the use of selective targeted agents and warrant interference at different stages for reducing the tumor burden effectively. Further, different etiological factors and risk habits can result in distinct genetic and epigenetic alterations, which may trigger different signaling pathways that impact development and progression of HNSCCs. A proof of principle is the constitutive activation of Ras/Raf/MAPK pathway due to *Ras *mutations in the areca quid chewing oral cancers, while in cancers associated with chronic tobacco exposure this pathway is likely to be activated downstream from EGFR activation. Another evidence of multiple aberrant pathways is the altered NFκB function leading to activation of STAT3 by an autocrine or paracrine mechanism initiated by IL-6 release, independent from EGFR. In addition, de novo/acquired chemoresistance comprises a significant problem in management of HNSCCs. The emerging data suggests Cancer Stem Cells (CSCs) may be responsible for acquired resistance to CT/RT in HNSCCs. Cancer stem cells is a subpopulation of cells that can self-renew and produce differentiated cells that form the bulk of the tumor [55]. It is proposed that the current HNSCC treatment regimens selectively kills the differentiated cancer cells producing tumor regression, but do not eliminate the cancer stem cells. Understanding the molecular signatures of HNSCC stem cells will define new targets for designing of novel therapeutic strategies.

Little attention has been paid to understanding the effect of human papillomavirus (HPV) on therapeutic response to targeted agents in HNSCC. It is being increasingly recognized that the molecular pathogenesis of HPV infected oropharyngeal SCC exhibits marked geographical variation [56] and is different from tobacco and alcohol associated HNSCC [7,13,57], so how can these biologically different tumors show the same response to targeted agents? In fact, HPV associated HNSCC show better prognosis than HPV negative tumors, though the molecular basis of improved prognosis is not clearly understood. Nevertheless, in future trials, evaluation of HPV status of HNSCC patients is likely to provide better insight into the outcome of clinical response to targeted agents.

### Future Strategies

The development of new biological agents should focus on inhibitors that are likely to hit multiple targets. Alternatively, combination of different agents that target distinct specific pathways is likely to inhibit the escape of tumor cells by alternate mechanisms leading to more effective disease control. But, the success of future clinical trials will depend upon (i) patient population and (ii) study design for assessment of response to therapy. Further, to evaluate the efficacy of these biological agents there is urgent need to identify novel biomarkers that can be used to accurately assess and individualize therapy.

#### Patient population selection

Phase II trials are often conducted on patients having advanced loco-regional disease or recurrent/metastatic HNSCC and have several limitations: (i) most patients have received RT/CT/CRT for the primary tumors, often develop multifactorial resistance and are less likely to respond to new agents effectively. This is best exemplified by the outcome of trials with cetuximab. In advanced HNSCC patients' refractory to platinum, cetuximab showed a response rate of 13%, while the response rate increased to 20% in patients who were stable on platinum therapy [10]. Hence testing of new agents as first line therapy is likely to show better clinical response than in recurrent/metastatic HNSCC patients; (ii) cumulative resistance observed in recurrent/metastatic HNSCC patients limits the generalization of clinical response to patients with early disease for target validation; (iii) feasibility of conducting translational research is hampered by the ethical constraints in obtaining tumor biopsies. A paradigm shift in design of phase II trials is proposed that enables evaluation of new compounds in pre-operative window setting. The collection of biopsies before treatment at the time of diagnosis, and after treatment, either at the time of surgery or before loco-regional therapy will permit assessment of predictive molecular markers and may help in identifying subgroups of patients most likely to respond to therapy (or develop primary resistance). In addition, these paired tumor specimens are likely to provide insights into the pharmacodynamic effects of novel agents, and their mechanism of action. Establishment of a data base documenting the specificities of the inhibitors and observed toxicities in Phase I and Phase II studies would provide a valuable resource for understanding whether there are particular cellular targets whose inhibition should be avoided.

#### Molecular markers for assessment of biological response

Evaluation of clinical response dependent on progression-based endpoints is likely to provide more realistic assessments of the anti-tumor activity of biological agents. Specific molecular markers may give a more objective evaluation of clinical response. To cite an example – pharmacodynamic tissue studies conducted on a phase I/II trial of erlotinib and cisplatin in patients with recurrent or metastatic HNSCC showed that high *EGFR *gene copy in tumor specimens may predict which patients are likely to respond to erlotinib, and decreased p-EGFR level in skin biopsies during therapy may represent a potential surrogate marker for improved clinical outcome. Multidimensional scaling (MDS) was shown to represent a novel way to evaluate these relationships between molecular markers and clinical outcome [58]. Molecular imaging or dynamic control enhanced CT/MRI may be used for measurement of intra-tumoral blood flow perfusion parameters to evaluate the clinical efficacy of anti-angiogenic agents.

There is a need for more predictive tumor models and better ways to monitor target inhibition in humans in a minimally invasive manner. As cell culture and animal models are severely limited in mimicking the development of human HNSCC, there is an urgent need to develop minimally invasive methods to discover and monitor biomarkers for evaluation of HNSCC in humans. New imaging modalities in conjunction with proteomic technologies may be investigated to monitor changes in signaling proteins and metabolites. Early detection technologies may allow us to diagnose and exterminate tumors prior to acquisition of survival capabilities that empower them with resistance to therapy.

In conclusion, in depth understanding of how the complex cellular signaling cascades and networks are reprogrammed in HNSCC and in the presence of mono-targeting inhibitors is vital to rational designing of combinations of inhibitors. Innovative trial designs and appropriate patient selection are critical for the success of new trials to translate molecular targeted therapies from the bench to the clinics.

## Abbreviations

5-FU: 5-Fluorouracil; ATP: Adenosine triphosphate; CRT: Chemoradiation; CSCs: Cancer Stem Cells; CT: Chemotherapy; ECOG: Eastern Cooperative Oncology Group; EGFR: Epidermal growth factor receptor; EGFRvIII: EGFR variant III; GPCR: G protein coupled receptor; HDACs: Histone deacetylases; HNSCCs: Head and Neck Squamous Cell Carcinomas; HPV: Human papillomavirus; IGF-1R: Insulin-like growth factor-1 receptor; PDGFR: Platelet derived growth factor receptor; RT: Radiotherapy; RTKs: Receptor tyrosine kinases; SCC: Squamous Cell Carcinoma; TKIs: Tyrosine kinase inhibitors; VEGF: Vascular endothelial growth factor; VEGFR: Vascular endothelial growth factor receptor.

## Competing interests

The authors declare that they have no competing interests.

## Authors' contributions

AM and RR wrote the manuscript. Both the authors read and approved the final manuscript.
